# Characterizing Non-covalent Protein Complexes Using Asymmetrical Flow Field-Flow Fractionation On-Line Coupled to Native Mass Spectrometry

**DOI:** 10.1021/acs.analchem.2c05049

**Published:** 2023-05-05

**Authors:** Iro Konstantina Ventouri, Wayne Chang, Florian Meier, Roland Drexel, Govert W. Somsen, Peter J. Schoenmakers, Bart de Spiegeleer, Rob Haselberg, Alina Astefanei

**Affiliations:** † Analytical Chemistry Group, Van ’t Hoff Institute for Molecular Sciences, University of Amsterdam, Science Park, 904, 1098 XH Amsterdam, The Netherlands; ‡ Division of BioAnalytical Chemistry, Amsterdam Institute of Molecular and Life Sciences, Vrije Universiteit Amsterdam, De Boelelaan 1085, 1081 HV Amsterdam, The Netherlands; § Centre of Analytical Sciences Amsterdam, Science Park, 904, 1098 XH Amsterdam, The Netherlands; ∥ Postnova Analytics GmbH, Rankinestraße 1, 86899 Landsberg, Germany; ⊥ Drug Quality and Registration (DruQuaR) Group, Department of Pharmaceutical Analysis, Faculty of Pharmaceutical Sciences, Ghent University, Ottergemsesteenweg 460, 9000 Ghent, Belgium

## Abstract

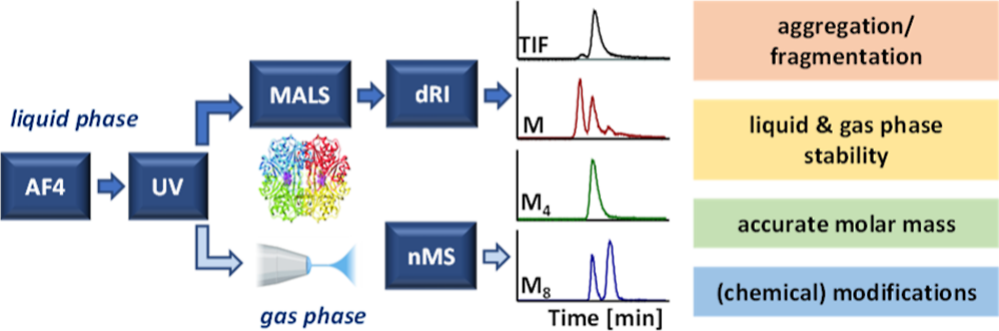

We report an online analytical platform based on the coupling of asymmetrical flow field-flow fractionation (AF4) and native mass spectrometry (nMS) in parallel with UV-absorbance, multi-angle light scattering (MALS), and differential-refractive-index (UV–MALS–dRI) detectors to elucidate labile higher-order structures (HOS) of protein biotherapeutics. The technical aspects of coupling AF4 with nMS and the UV–MALS–dRI multi-detection system are discussed. The “slot-outlet” technique was used to reduce sample dilution and split the AF4 effluent between the MS and UV–MALS–dRI detectors. The stability, HOS, and dissociation pathways of the tetrameric biotherapeutic enzyme (anticancer agent) l-asparaginase (ASNase) were studied. ASNase is a 140 kDa homo-tetramer, but the presence of intact octamers and degradation products with lower molecular weights was indicated by AF4–MALS/nMS. Exposing ASNase to 10 mM NaOH disturbed the equilibrium between the different non-covalent species and led to HOS dissociation. Correlation of the information obtained by AF4–MALS (liquid phase) and AF4–nMS (gas phase) revealed the formation of monomeric, tetrameric, and pentameric species. High-resolution MS revealed deamidation of the main intact tetramer upon exposure of ASNase to high pH (NaOH and ammonium bicarbonate). The particular information retrieved from ASNase with the developed platform in a single run demonstrates that the newly developed platform can be highly useful for aggregation and stability studies of protein biopharmaceuticals.

## Introduction

Protein biopharmaceuticals encompass a diverse group of structures, including therapeutic enzymes, monoclonal antibodies (mAbs), fusion proteins, hormones, and cytokines derived from living cells.^1^ The biological and pharmacological functions of biopharmaceuticals are highly reliant on their well-defined three- and four-dimensional structures.^1^ Even slight changes in this so-called higher order structure (HOS) can have a dramatic impact on product quality and efficacy, including the generation of unwanted immunogenic reactions.^2^ As a result, HOS preservation is a major concern in the manufacturing of protein biopharmaceuticals.^3,4^ However, the large size and complex structure of protein biotherapeutics pose a difficulty of their characterization. Additionally, preservation of the HOS during sample preparation and/or analysis is an immense analytical challenge. There is no single analytical technique that reliably reveals the detailed structural complexity of a biopharmaceutical’s HOS.^5,6^ Therefore, there is a continuous quest for more advanced analytical tools for structural characterization. In this context, the development of analytical platforms based on synergistic combinations of separation techniques with high-resolution mass spectrometry, light scattering, and/or fluorescence detection can produce essential and representative information on the HOS of biopharmaceuticals.

Native mass spectrometry (nMS) has seen tremendous development over the past few decades for the characterization of protein biopharmaceuticals.^7−9^ nMS allows preservation of the structure of complexes during electrospray ionization (ESI), enabling accurate determination of their molar mass and stoichiometry. Even complexes held together by weak non-covalent interactions can be preserved during ESI.^9^ However, sample preparation for nMS usually requires the manual exchange of buffer and/or salt with volatile additives. This step is time-consuming, labor intensive and may also cause alterations to the protein, such as aggregation, precipitation, or even chemical modification.^10^ Furthermore, the complexity of biological samples and the presence of highly similar protein species often lead to ionization suppression and convoluted mass spectra, making data interpretation challenging. Consequently, comprehensive characterization of biotherapeutics often benefits from efficient analytical separations prior to detection by nMS.

Combinations of non-denaturing separations, such as size-exclusion chromatography (SEC), hydrophobic-interaction chromatography, ion-exchange chromatography, and capillary electrophoresis, with nMS have been established.^11^ SEC is most often used to separate size variants of proteins arising from truncation, aggregation, or oligomerization.^12−14^ However, SEC–nMS is commonly performed under high-ionic-strength conditions in order to prevent adverse interactions between the stationary phase and protein molecules, at the cost of the ionization efficiency of analytes. Volatile ammonium-based salts facilitate online SEC–nMS but may not prevent unwanted protein–column interactions as effectively as phosphate buffers.^15^ In addition, high-shear conditions, denaturing mobile phases, and dilution can cause changes in the conformation of the protein and its aggregates.^16^

Asymmetrical flow field-flow fractionation (AF4) is a size-based separation method that allows the separation and quantification of large (bio)-macromolecules, particles, and aqueous polymers.^17−19^ AF4 is often used coupled to a multi-detector system including UV-absorbance, differential-refractive-index (dRI), and multi-angle light-scattering (MALS) detectors. The separation in AF4 takes place in an open channel without a stationary phase or a packing material, offering indisputable advantages. An ultrafiltration membrane of a desired material and molecular weight cut-off determine the lowest size of sample components that are retained in the channel. Mechanical and/or shear stress and the risk of filtering effects are minimal.^20^ The carrier-liquid composition can be tailored to preserve protein structures and maintain biological activity while maximizing recoveries. Aqueous buffers of low ionic strength (<100 mM) are often used in AF4,^20^ which is particularly attractive for coupling with nMS.^15,21^

Few studies using either a miniaturized AF4 channel or a hollow-fiber-flow-field-flow fractionation (HF5) system show the direct coupling to ESI-MS for the identification of model proteins (up to 78 kDa),^22,23^ and lipids and lipoproteins from plasma samples.^24−26^ In these studies, stable proteins of low molecular weight were used to primarily demonstrate the potential of sample desalting and clean-up when using AF4 as a (pre)-separation before MS. However, the full potential of AF4 coupled to both nMS and MALS for in-depth structure elucidation of the HOS of protein biotherapeutics has not yet been realized. Combining liquid-phase light scattering and ESI-MS data can provide new insights into the stability, composition, and HOS of biopharmaceuticals. Separating compounds with AF4 and detecting them with MALS and nMS in parallel can identify species across a broad size range in a single run. This enables a direct correlation between the physico-chemical properties and structural information of eluting species. To achieve this, a regular AF4 channel is crucial to avoid excessive band broadening caused by the relatively large (cell) volumes of liquid-phase detectors.

In this work, we have been exploring the parallel coupling of AF4 to nMS and UV–MALS–dRI, to achieve a detailed characterization of the HOS of protein biotherapeutics. We used the tetrameric biotherapeutic enzyme (anticancer agent) l-asparaginase (ASNase) as a model compound and exposed it to various stress conditions such as pH, temperature, and agitation. ASNase may exist in monomeric (one protein unit; 3.5 × 10^4^ g/mol), tetrameric (1.4 × 10^5^ g/mol), and higher-order oligomeric structures (above 2.5 × 10^5^ g/mol), covering a broad molar mass range. The most active and abundant ASNase species is thought to be a tetramer composed of four identical monomeric subunits. We explored quantitative correlations between the structural information obtained from the liquid phase (AF4 and UV–MALS–dRI) and from the gas phase (nMS), shedding light on different denaturation pathways of ASNase, on the dynamic equilibria between the various oligomeric assemblies, and on the stabilities of the various species. The technical aspects of coupling analytical AF4 to nMS have also been investigated and discussed.

## Materials and Methods

### Asymmetrical Flow Field-Flow Fractionation (AF4–UV–MALS–dRI)

All the chemicals and solutions used in this study can be found in the Supporting Information S1.0. Experiments were performed using an AF2000 MultiFlow FFF system (Postnova Analytics, Landsberg/Lech, Germany), coupled to an SPD-20A UV/vis absorbance detector operated at 280 nm (PN3212; Shimadzu, Kyoto, Japan), a MALS detector (PN3621), and a refractive index (dRI) detector (PN3150) at a working temperature of 40 °C. The Smart Stream Splitter module (PN1650) was used to facilitate the slot-outlet (SO) technique, allowing splitting of the outlet eluent stream leaving the FFF channel.^27,28^ The dimensions of the AF4 block were 335 mm length × 60 mm width. The separation channel had a tip-to-tip length of 277 mm, an initial width of 20 mm, and a final width of 5 mm, with a 350 μm spacer thickness. The analytical AF4 channel is commercially available by Postnova Analytics (AF-28AN). A 10 kDa molecular-weight cut-off membrane prepared from regenerated cellulose (Postnova) was used as the accumulation wall. Data acquisition was carried out by AF2000 control software version 2.1.0.1 (Postnova). The molar mass and weight-average molecular weight (*M*_w_) were calculated using the Zimm model and a refractive index increment (d*n*/d*c*) of 0.185 [mL g^–1^]. In these calculations, the angles of 7, 12, 20 and 158, 164° were excluded, as their signal-to-noise ratios were too low for accurate measurement.

Calibration of the concentration detectors (UV, dRI) and the size-specific optical detector (MALS) was performed using bovine serum albumin (66 kDa) in concentrations of 1 and 5 mg/mL, respectively. Normalization of all the angles of MALS was performed using a 10 mg/mL solution of polystyrene-sulfonate sodium salt (PSS; molecular weight 63.9 kDa; Postnova Analytics). For the calibration of the detectors and normalization of the various angles, a 0.150 M sodium chloride solution was used. Recoveries were determined from the ratios of the UV peak areas of the separated oligomeric species while applying cross flow, divided by the area obtained when the sample was eluted through the channel at the same outlet flow without cross flow (*F*_c_ = 0).^29^ Highly retained sample and higher-order structures eluting during the rinsing step (*F*_c_ = 0) were not included in the recovery estimation. Relative peak areas of the various species of ASNase were estimated based on the peak area of individual peaks divided by the total peak area.

### Analysis of ASNase by AF4–UV–MALS–dRI

Initial AF4 measurements were carried out using a phosphate-based carrier liquid (PB; see Section S1.0). Sample injection was performed at an injection flow (*F*_inj_) of 0.20 mL/min for 5 min using a cross-flow rate (*F*_c_) of 3.0 mL/min and a subsequent focus flow rate of 3.30 mL/min. The detector flow rate (*F*_out_) was set at 0.50 mL/min. After focusing and during elution, *F*_c_ was kept constant at 3 mL/min for 20 min, followed by a linear decay down to *F*_c_ = 0.1 mL/min during 19 min. *F*_c_ was then kept constant at 0.1 mL/min for 10 min. Finally, in during the rinsing step, *F*_c_ was turned to zero and a laminar flow was maintained through the channel (*F*_out_ = 0.5 mL/min) during 5 min.

### AF4–nMS Method

For the coupling of AF4 to nMS, a 10 mM ammonium acetate (pH 6.8) carrier liquid was used. A flow splitter was introduced immediately after the UV detector in order to divide the flow between MALS–dRI (0.2 mL/min) and nMS (0.2 mL/min). A schematic overview of the set-up is presented in Figure S1. Sample injection was performed at an injection-flow rate (*F*_inj_) of 0.20 mL/min for 4 min using a cross-flow rate (*F*_c_) of 3.0 mL/min and a subsequent focus flow rate of 3.30 mL/min. The laminar channel flow rate was 0.50 mL/min, and the slot-flow rate controlled by a separate module based on the SO technique^27,28^ was set at 0.10 mL/min, maintaining the detector-flow rate (*F*_out_) at 0.4 mL/min. The flow program was identical to the one described above for the AF4–UV–MALS–dRI analysis.

### Mass Spectrometry

AF4–nMS experiments were performed using a Q-Exactive Plus Hybrid Quadrupole-Orbitrap mass spectrometer (Thermo Fisher Scientific, Bremen, Germany), equipped with an ESI source operated in positive-ionization mode. The instrument was controlled by Xcalibur software version 3.0 (Thermo Fisher Scientific). Mass analysis of the proteins was performed in the *m*/*z* range from 2000 to 8000. MS conditions were as follows: spray voltage, 3.5 kV; capillary temperature, 275 °C; in-source collision energy (is-CID), 20.0 eV; sheath gas and auxiliary gas flow rate, 15 and 5 units; respectively, and auxiliary gas heater temperature, 175 °C. The automatic gain control target value was set to 3 × 10^6^ and the resolution to 17,500. The selected resolution settings were found to provide the best sensitivity and highest accuracy for mass assignment. Data analysis was performed using FreeStyle 1.6 software (Thermo Fisher Scientific). Deconvolution of the recorded protein mass spectra was carried out using the intact protein analysis option in the BioPharma Finder 4.0 application (Thermo Fisher Scientific). For the deconvolution of the mass spectra also the UniDec program (University of Arizona, Phoenix, AZ, USA) was used.^30^

## Results and Discussion

### AF4–MALS of ASNase

The biopharmaceutical enzyme ASNase presents itself predominantly as a non-covalent tetrameric complex of about 1.4 × 10^5^ g/mol in an aqueous solution.^31^ It also exhibits residual monomeric species (approximately 3.5 × 10^4^ g/mol) as well as HOS (above 2.7 × 10^5^ g/mol). Moreover, ASNase expressed in *Escherichia coli* does not exhibit naturally occurring post-translational modifications,^32^ while deamidation may be induced during the manufacturing and storing processes.^33^ Therefore, ASNase was selected as a highly relevant and suitable protein to study and demonstrate the possibilities of AF4–nMS.

An AF4–UV–MALS–dRI method was developed that provides separation of ASNase and its main size variants. Critical AF4 parameters (cross-flow rate, focusing time, and injected amount) were evaluated using 50 mM phosphate buffer and 50 mM sodium chloride at pH 6.9 as a carrier liquid. The best separation was obtained using a constant cross-flow rate (*F*_c_) of 3 mL/min, an outlet flow rate (*F*_out_) of 0.5 mL/min, and a focusing time of 4 min, while the focusing flow (*F*_Foc_) was set at 3.30 mL/min. Figure S2 shows the resulting fractogram under these conditions. Three well-defined peaks can be observed. Based on the MALS data, these could be assigned to tetrameric (M_4_; main peak, 1.4 × 10^5^ g/mol), octameric (M_8_; 2.7 × 10^5^ g/mol), and dodecameric (M_12_; 4.2 × 10^5^ g/mol) ASNase (Figure S2). After the dodecameric species, a heterogeneous zone of HOS was observed, which had molecular weights ranging from 5.0 × 10^5^ g/mol up to about 8.0 × 10^5^ g/mol. Due to low signal intensity and peak overlap, no clear structures could be assigned. With this method, satisfactory recovery (>85%) and excellent repeatability of elution time (RSD < 1.2%, *n* = 3) and peak area (RSD < 5%, *n* = 3) were obtained. The relative peak areas for tetramer, octamer, and dodecamer of 74%:18%:7% were consistently found between analyses and various samples. Very minor amounts of ASNase monomer (M; less than 1% of the total sample) were also detected. Due to this low amount of the monomer species, MALS did not provide an accurate MW. Hence, identification of the ASNase monomer was done based on mass spectrometric data (see Figure 2A).

**Figure 1:**
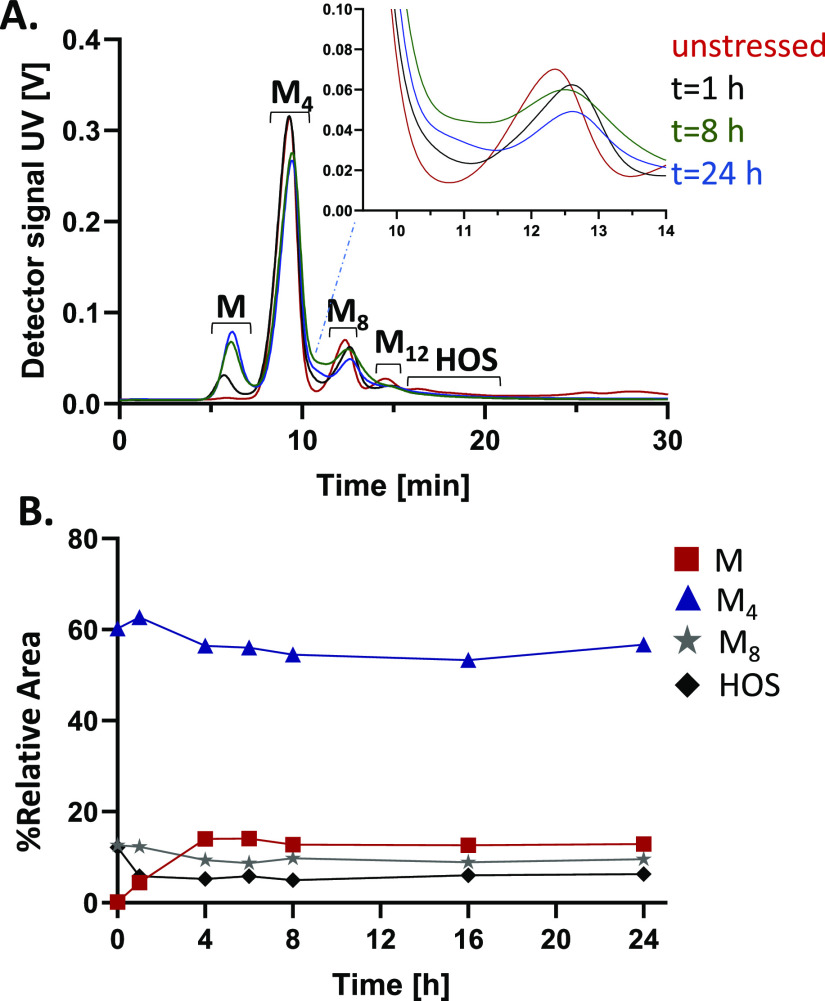
(A) Fractograms obtained from AF4–UV–MALS analysis of unstressed ASNase (red trace) and ASNase exposed to 10 mM NaOH for 1 h (black trace), 8 h (green trace), and 24 h (blue trace). (B) Relative peak areas of ASNase monomer (M), tetramer (M_4_), octamer (M_8_), and higher-order structures (M_12_ + HOS) obtained after different times of exposure to 10 mM NaOH. For AF4 conditions, see Figure S2.

Subsequently, ASNase dissolved in phosphate buffer was exposed to various stress conditions, such as (i) elevated temperature (53 °C, 2–24 h), (ii) agitation (vortex mixer, 1500 rpm, 30 min), and (iii) high pH (about 12) using 10 mM NaOH (1–24 h).^34,35^ Agitation did not show a significant effect on aggregation or dissociation (Figure S3A). Elevated temperature resulted in the formation of approximately 2% (relative peak area) of ASNase monomer in comparison to the unstressed ASNase (Figure S3B). The most significant effect was observed when ASNase was exposed to 10 mM NaOH (Figure 1). Whereas in the unstressed sample almost no monomer was observed (<1% in peak area), it increased with the duration of the high-pH exposure (Figure 1A). Simultaneously, a clear decrease of the peaks corresponding to the tetramer, octamer, and dodecamer was observed.

By plotting the relative peak area of all the species against the duration of high-pH exposure (Figure 1B), it becomes evident that the oligomers dissociate (peak areas are decreasing) and contribute to the formation of lower-molecular-weight oligomers, i.e., monomeric ASNase. This mainly happens during the first 4 h, after which a plateau appears to be reached. This may indicate that the larger species are less stable than the tetrameric form. However, it should be noted that the dissociation pathways of the HOS to the formation of lower-molecular-weight oligomers cannot be fully discerned solely based on this data.

A closer examination of the fractograms indicated that in the NaOH-exposed samples, unresolved species between the tetramer (M_4_) and octamer (M_8_) are present (Figure 1A, enlarged area). The exact source of these unresolved species is not straightforward to highlight. The original dynamic equilibrium between the various oligomeric species is disturbed in the channel during the separation. The continuous re-establishment of the equilibrium during the separation may lead to band broadening. Additionally, there is an indication of dissociation of the octameric and the higher-order oligomers into lower-molar-mass species that elute between the tetramer and the octamer (Figure 1B).

The cross-flow rate (3.0, 4.5, and 5.5 mL/min; Figure S4A) and the injected amount (between 15 and 150 μg; Figure S4B) were varied in order to improve the separation between the various oligomeric species. Low injected amounts (not larger than 30 μg) eliminated the overloading effects, yielding better resolution between the various oligomeric species. A cross-flow rate between 3 and 4.5 mL/min (recovery values above 80% in both cases) sufficed to separate the main oligomeric species (monomer, tetramer, and octamer). A cross-flow rate of 5.5 mL/min resulted in a broader tetramer peak, indicating protein–protein interactions, possibly due to a smaller mean layer thickness under these conditions. Hence, flow rates between 3 and 4.5 mL/min provided better separation and were used for further experiments.

The molar mass of these not well-resolved species could not be extracted from the MALS signal regardless of the AF4 elution conditions (Figures S5 and S6). Although accurate values were obtained for the monomeric and tetrameric ASNase (3.5 × 10^4^ and 1.4 × 10^5^ g/mol, respectively), the later eluting species showed molar masses lower than expected (Figure S6). These values could not be assigned to defined oligomeric structures (molar masses of 7.2 × 10^4^ and 1.7 × 10^5^). The molar mass estimates for these species could have been affected by the low signal intensity and, subsequently, the increased noise level in the MALS signals, along with the low separation resolution.

### AF4–nMS Hyphenation: Critical Parameters and Detection of Protein Complexes

To obtain more structural information, the addition of online nMS, next to MALS, can be very useful. To allow direct coupling of AF4 with nMS, the non-volatile phosphate-based carrier liquid had to be exchanged for a volatile alternative. Ammonium acetate is commonly used for nMS, ensuring that proteins stay in their native conformation during both separation and MS detection when used in low concentrations.^15^ Using 10 mM ammonium acetate (pH 6.8) instead of 50 mM phosphate and 50 mM sodium chloride in the carrier liquid resulted in a comparable fractogram (Figure S7). A slight shift toward earlier elution times was observed with ammonium acetate. This is thought to be caused by the lower diffusivity of the protein under lower ionic strength conditions and/or due to the different surface ζ potentials of the protein and the membrane, resulting in different equilibrium heights within the channel during the focusing. However, this did not impact the analytical performance. By using 10 mM ammonium acetate, the recovery rate exceeded 87%, and the separation efficiency was comparable with a resolution (*R*_s_) between tetramer and octamer of 1.8 and 2.0 for the acetate-based and phosphate-based carrier liquids, respectively. The peak area ratios obtained with the two carrier liquids for tetramer–octamer–dodecamer were also similar, i.e., 74:18:8 and 77:16:7, respectively, for phosphate and acetate.

To allow an efficient coupling of AF4 to nMS, a 1:1 flow splitter was placed after the UV detector (Figure S1). This resulted in equal portions of the AF4 effluent to be directed to the nMS instrument and MALS–dRI detector. Lowering the flow to the mass spectrometer improves the ionization efficiency, allows milder interfacing conditions (lower temperatures) to be used, and reduces source contamination. As the lower flow toward the optical detectors negatively impacts the sensitivity, the SO technique was applied.^27,28^ With SO technology, only the sample-enriched part of the channel-outlet flow (close to the membrane) is sent to the detectors. The slot flow was set to 0.1 mL/min, which resulted to a *F*_out_ of 0.4 mL/min. The use of the SO technique increased the overall sensitivity by approximately a factor of 1.5. Using the 1:1 split in combination with the SO technology, the flow rate toward nMS was 0.2 mL/min, and a similar flow rate was sent to the optical detectors.

**Figure 2:**
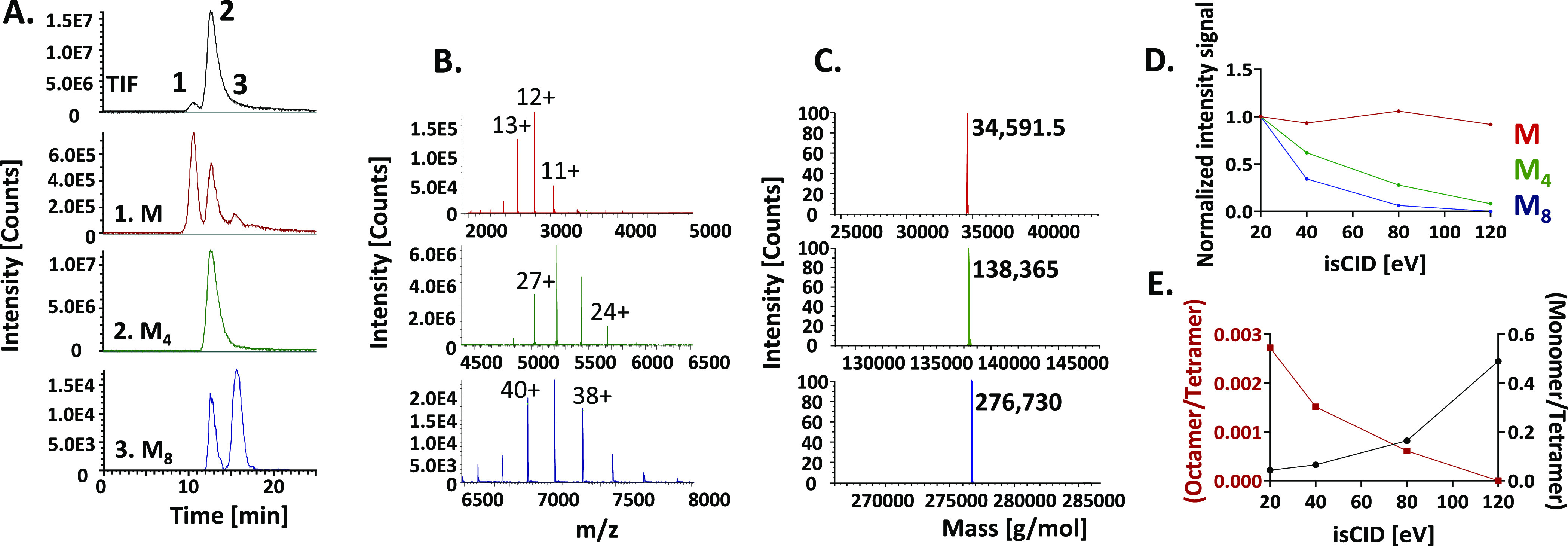
AF4–nMS of unstressed ASNase. (A) TIF and IEFs based on the characteristic *m*/*z* values of the monomeric (M), tetrameric (M_4_), and octameric (M_8_) species. (B) Average mass spectra obtained for each peak; (C) deconvoluted mass spectra of the various species (M, M_4_, and M_8_); A–C: isCID energy = 20 eV. (D) Impact of isCID energy on the signal intensity of the three species (M, red; M_4_, green; M_8_, blue). (E) Impact of isCID energy on the monomer–tetramer (black) and octamer–tetramer (red) ratios.

For the initial online AF4–nMS experiments of ASNase, an MS method facilitating large-molecule detection was selected (for details, see Materials and Methods); the mass range was set from *m*/*z* 2000 up to 8000 (instrument maximum). An isCID energy of 20 eV was applied during the method acquisition. In Figure 2A the obtained total-ion fractogram (TIF) and a few selected extracted-ion fractograms (EIFs) are depicted. At the apex of the main peak, a mass spectrum centered around *m*/*z* 5500 was obtained, of which the signals could be assigned to the [M_4_ + 23H]^23+^ to [M_4_ + 28H]^28+^ charge states of ASNase, where M_4_ is the tetramer mass (Figure 2B). The resulting protein molecular weight after deconvolution was 138,365 Da (Figure 2C). In addition, low-intensity peaks were observed for the ASNase monomer and the octamer. Deconvolution of the mass spectra (Figure 2B,C) led to molecular weights of 34,591.5 and 276,730 Da, respectively. ASNase derived from bacterial sources (*E. coli*) has a theoretical mass for the monomeric species of 34,591.6 Da (average mass, based on known sequence^36^ and one disulfide bridge between Cys_77_ and Cys_105_,^37,38^Figure S8), leading to theoretical average masses of 138,366.2 and 276,732.5 Da for the tetramer and octamer, respectively. Clearly, the AF4–nMS system allows proper detection of these large non-covalent protein complexes. Note that the observed monomodal charge-state distributions have a maximum charge state of [M + 12]^12+^, [M_4_ + 26]^26+^, and [M_8_ + 39]^39+^ for the native monomer, tetramer, and octamer, respectively. This corresponds to the native/non-denatured conformation of the species of ASNase with the expected maximum charge states of [M + 14H]^14+^, [M_4_ + 29H]^29+^, and [M_8_ + 41H]^41+^, respectively.^39^

A more-detailed examination of the EIFs recorded for the three species (Figure 2A) reveals some interesting aspects. First, discrete signals of monomer are detected at the elution times of the tetramer and the octamer. This indicates that a small fraction (<1%) of the tetramer and octamer dissociates in the gas phase into monomeric species. This estimation assumes equal ionization efficiency of the species and recognizing that one tetramer dissociates into four monomer units. At the elution time of the tetrameric species, a signal for the octameric ASNase was also observed. This may be explained by the association of tetramers during the ESI process, promoted by the high concentration of tetrameric ASNase attained during desolvation.^40,41^ Assuming equal ionization efficiency, the octamer signal amounts to about 5% of the total tetramer signal. Eliminating the isCID (0 eV) resulted in a similar gas-phase dissociation pattern (data not shown). However, the sensitivity was compromised, especially for the higher-order oligomers (octamers), likely due to insufficient desolvation of the protein being electrosprayed from a purely aqueous solution. Variation of the spray voltage and nebulization gas pressure did not change the results. Increasing the isCID energy, on the other hand, showed a clear effect (Figures S9 and 2D). Whereas the monomer signal remained unchanged, the signal intensity of the tetramer decreased to <10% when the isCID energy was raised from 20 to 120 eV. At high isCID voltages, the octamer was no longer detectable, but in the EIF of the tetramer, a second signal was observed at the elution time of the octamer. This is most likely due to gas-phase dissociation, where the octamer seems more susceptible than the tetramer, which is in line with the observation that the octamer is the less-stable conformation (see discussion of Figure 1B). To investigate that further, the relative (%) monomer-to-tetramer and octamer-to-tetramer ratios (Figure 2E) were plotted based on the relative peak area of the respective species extracted using their distinct *m*/*z* values. Figure 2E shows that ratios change depending on the isCID voltage. Raising the isCID leads to an increase in the monomer-to-tetramer ratio from around 4% to close to 50%, whereas the octamer-to-tetramer ratio decreases from 0.3% to <0.05%. As ionization efficiencies for these different ASNase structures are presumably not the same, a direct comparison with the quantitative data from the optical detectors cannot be made reliably. However, based on the peak ratios obtained with the UV detector (i.e., 74:18:8 for tetramer, octamer, and dodecamer, respectively) and the amount of monomer being not detectable, the ions observed at the lowest isCID voltage (20 eV) resemble the sample composition most accurately while also yielding the highest detection sensitivity. Notably, due to the AF4 separation, true and method-generated species were effectively discerned.

### AF4–nMS Analysis of Stressed Samples

After establishing an AF4–nMS method for the separation and detection of various natural ASNase oligomers under near-native conditions, the applicability toward the identification of species formed during high-pH exposure (dissolved in 10 mM NaOH) was investigated. The experiments were performed using 10 mM ammonium acetate (pH 6.8) as the carrier liquid and an isCID voltage of 20 eV (Figure S10). The resulting fractograms for ASNase stressed for 8 h are shown in Figure S6. For the construction of species-specific EIFs, a set of unique *m*/*z* values was generated for the monomer and each of the multimeric species up to the decamer (Table S1).^42^ Nonamers and decamers are very close to the upper limit of the mass spectrometer’s measurement range and were not detected. Larger multimers were not included as their unique *m*/*z* values were outside the measurement range. When analyzing NaOH-stressed ASNase, monomeric, dimeric, trimeric, tetrameric, pentameric, and octameric ASNase species were detected (Figure S10); no other aggregates were observed. The three main species found in the unstressed sample—monomer, tetramer, and octamer—were also clearly observed in the pH-stressed sample. A distinct signal for the dimer and trimer is found at the elution time of the monomer. Most likely, this is the result of gas-phase multimer formation due to the high concentrations of monomers formed. The more-interesting region in the fractogram is between the tetramer and octamer, where detailed information could not be obtained with MALS detection (see discussion of Figures 1 and 2). In this region, mainly dimeric and pentameric ASNase were detected (Figure S10). The pentamer showed a low-intensity but clearly defined peak and is most probably present in the sample and not formed in the ESI process. The exact mechanism of pentamer formation is not clear. In contrast, the signal observed for the *m*/*z* values of the dimeric ASNase is broad and spans the entire time window from tetramer to octamer and beyond. Obviously, dimeric ASNase species (approx. 6.9 × 10^4^ g/mol) cannot elute after tetrameric ASNase in AF4. This suggests that dissociation events occur in the liquid phase, as indicated by the loss of resolution between the higher oligomeric species. This is also in line with the MALS data that show—on average—lower molecular weights in solution than expected. From the EIF, dimeric species are detected in the region where the higher-order oligomers elute. This suggests additional gas phase dissociation of unstable oligomeric species. In this context, the light-scattering and MS data are nicely in agreement. However, AF4–nMS provides a much more detailed insight into the subunits that take part in the dynamic equilibria.

**Figure 3:**
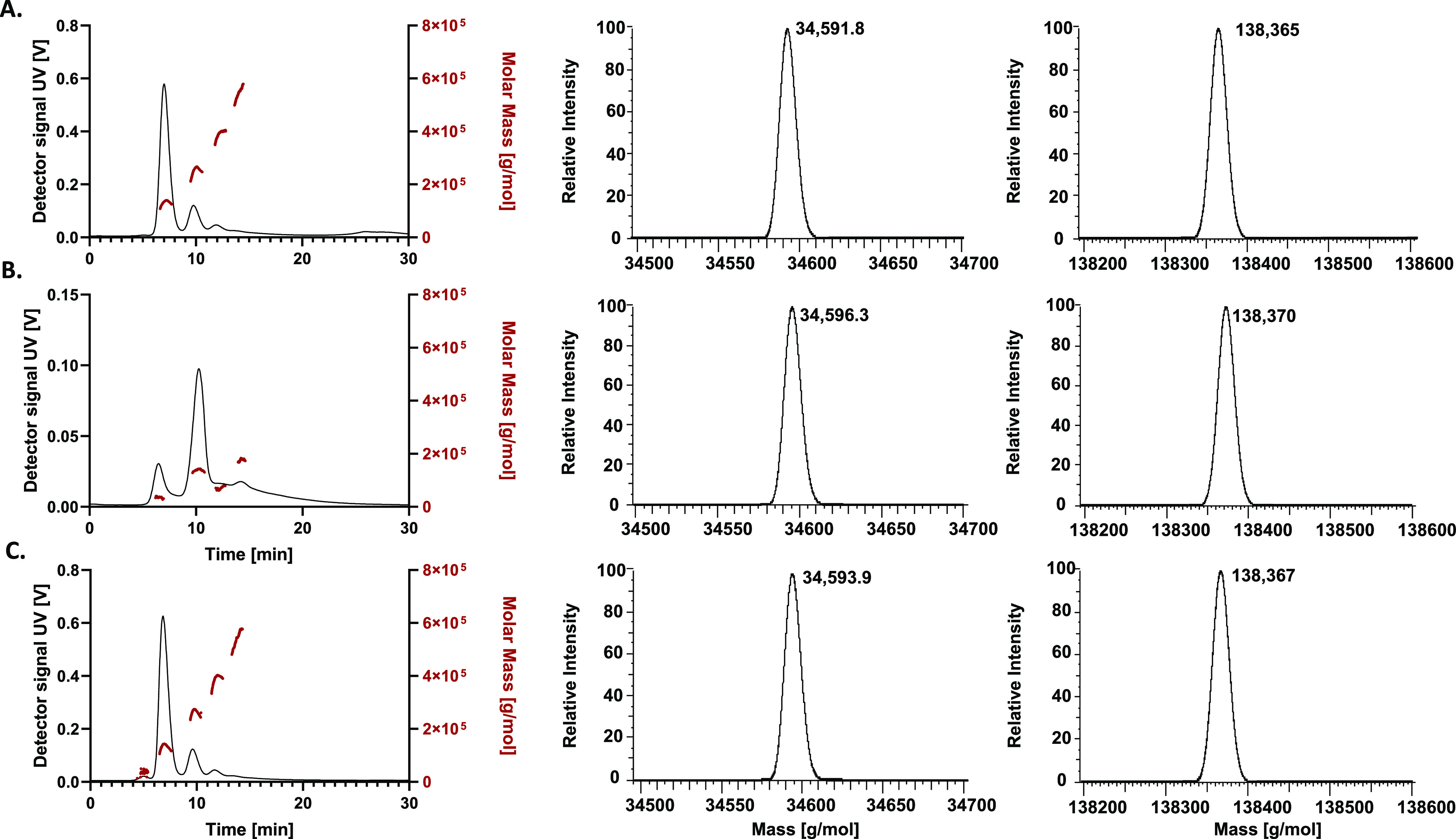
AF4–UV–MALS–dRI fractograms of (A) unstressed ASNase and of ASNase exposed to (B) 10 mM NaOH for 8 h, and (C) 10 mg/mL NH_4_HCO_3_, 37 °C for 24 h. Left, UV fractograms; right, deconvoluted mass spectra of the observed monomers (M) and tetramers (M_4_). Carrier liquid, 10 mM ammonium acetate (pH 6.8). Top and bottom fractograms: elution with constant *F*_c_ = 3.0 mL/min and *F*_out_ = 0.4 mL/min; injected mass, 30 μg. Middle fractogram: *F*_c_ = 4.5 mL/min and *F*_out_ = 0.4 mL/min; injected mass, 20 μg.

Focusing on the monomer and tetramer (Figure 3), deconvoluted masses of 34,596.3 Da (measurement error is ±0.4 Da, *n* = 3) and 138,370 Da (±0.8 Da, *n* = 3) were obtained, respectively, in the NaOH-stressed sample. Compared to the unstressed sample—yielding average masses of 34,591.5 ± 0.3 and 138,365 ± 0.1 Da (*n* = 3)—this represents an average increase of 5 Da for both species (compare 3A and 3B). Solely based on mass deviation, it is impossible to accurately assign the root cause of the shift in molecular weight. The isotopically unresolved mass envelope of this high-mass molecule is broad (approximately 25 Da at full width at half-maximum), limiting the information provided.^43^ However, based on a previous study in which ASNase was intentionally deamidated, it is very plausible that this mechanism is the cause of the mass shift.^44^ As deamidation causes a mass increase of 0.984 Da, the observed mass differences indicate an estimated average occurrence of 5 deamidations for the monomer and tetramer, respectively. Since the ASNase monomer has many asparagine and glutamine residues^45,46^ the 8 h exposure to NaOH most probably resulted in the modification of a part of these residues only. Note that the mass difference is above the limit of the mass accuracy of the instrument (deviation is 30–120 ppm, accuracy is approximately 1 ppm within a run), confirming the trueness of the deviation. Repeated analyses of both the stressed and unstressed samples also show that the difference is always toward a higher molecular weight and is statistically significant (*t*-test, α = 0.05; *p* < 0.0001 and *p* = 0.0004 for the monomer and tetramer, respectively).

To verify whether these small mass differences can be reliably detected and could be the result of deamidation, an additional experiment was performed. ASNase was incubated in 10 mg/mL ammonium bicarbonate (NH_4_HCO_3_; approx. pH 8.5) at 37 °C for 24 h and analyzed by AF4–MALS–nMS (Figure 3C). Incubating ASNase under these conditions is known to induce deamidation while keeping the native structure intact.^44^ Indeed, the obtained fractogram and light-scattering data are nearly identical to those obtained for unstressed ASNase (only an increase of approximately 1.5 % of the relative peak area of monomer was observed, Figure S11), confirming that exposure to bicarbonate does not yield dissociation of multimeric species. However, a difference is observed in the deconvoluted mass spectra. The deconvoluted mass of the monomer and tetramer are now 34,593.9 ± 0.5 and 138,367 ± 0.7 Da (both *n* = 3), both being significantly increased compared to the unstressed sample (*t*-test, α = 0.05; *p* = 0.0067 and *p* = 0.008 for the monomer and tetramer, respectively). This confirms that even small deviations in molecular weight—such as those caused by deamidation—can be picked up using AF4–ESI–nMS.

## Conclusions

The online coupling of AF4 with multiple detectors, including MALS and nMS, is reported for the characterization of intact non-covalent complexes of the biopharmaceutical ASNase under native conditions. The direct coupling of AF4 and nMS provides a number of advantages. The versatility of AF4 with respect to the carrier-liquid composition allowed the use of a low-ionic-strength volatile salt (10 mM ammonium acetate), resulting in excellent ESI compatibility while minimizing chances of sample degradation. Notably, eluent conditions (i.e., higher ionic strength, pH, and volatile salt) and flow rate split-ratio can be easily adapted in this platform. Although not critical in the current work, we have observed that these parameters impact the separation and gas phase transition of other proteins. nMS enabled the identification of intact protein species up to 2.7 × 10^5^ g/mol (ASNase octamer), providing valuable insights on the stability of various oligomers. The limiting factor for the detection of higher-molecular-weight species was the achievable measurement range of the mass spectrometer. Correlation of information obtained in the liquid (MALS detection) and gas phase (nMS) was useful to unravel the degradation pathways of ASNase. While AF4 coupled to MALS–dRI might not fully resolve various species after exposure to stress conditions, the additional separation and resolution provided by nMS reveal accurate masses and insights on the protein complex’s stability. Upon exposure to NaOH, dissociation of higher-order oligomers into monomers, tetramers, and pentamers was observed. Benefiting from the accuracy and precision of the mass spectrometer, ASNase was also shown to be prone to deamidate when exposed to higher pH (NaOH and ammonium bicarbonate). The applied optical and concentration detectors allowed relative quantitation of the protein species; ESI-MS is less suited for this purpose. Overall, the AF4–UV–MALS–nMS platform provides useful structural information of the labile HOS of protein biotherapeutics. Correlating the obtained information from both liquid and gas phase analysis facilitates the gain of insights into protein complex dissociation and aggregation pathways.

## Supporting Information Available

Additional experimental details, fractograms, extracted-ion-fractograms and information on the chemicals and materials, and a schematic representation of the platform (PDF)
