# Supplementary material for: Characterizing Non-covalent Protein Complexes Using Asymmetrical Flow Field-Flow Fractionation On-Line Coupled to Native Mass Spectrometry
Source: Anal Chem. 2023 May 5;95(19):7487–94. doi: 10.1021/acs.analchem.2c05049 (PMC10193367; doi:10.1021/acs.analchem.2c05049)
Supplement: Supplementary file 1 — ac2c05049_si_001.pdf [file ac2c05049_si_001.pdf]

Supporting Information

Characterizing non-covalent protein complexes using asymmetrical flow field-flow fractionation on-line coupled to native mass spectrometry

Iro Konstantina Ventouri[1,3*], Wayne Chang[1], Florian Meier[4], Roland Drexel[4], Govert W. Somsen[2,3], Peter J. Schoenmakers[1,3], Bart de Spiegeleer[5], Rob Haselberg[2,3], Alina Astefanei[1,3*]

1. University of Amsterdam, van 't Hoff Institute for Molecular Sciences, Analytical Chemistry Group, Science Park, 904, 1098 XH Amsterdam, The Netherlands
2. Vrije Universiteit Amsterdam, Amsterdam Institute of Molecular and Life Sciences, Division of BioAnalytical Chemistry, De Boelelaan 1085, 1081 HV Amsterdam, The Netherlands
3. Centre of Analytical Sciences Amsterdam, Science Park, 904, 1098 XH Amsterdam, The Netherlands
4. Postnova Analytics GmbH, Rankinestraße 1, 86899 Landsberg, Germany
5. Drug Quality and Registration (DruQuaR) Group, Department of Pharmaceutical Analysis, Faculty of Pharmaceutical Sciences, Ghent University, Ottergemsesteenweg 460, 9000 Ghent, Belgium

*corresponding authors

MSc. I. K. Ventouri, ventouri.iro@gmail.com

Dr. A. (Alina) Astefanei, a.astefanei@uva.nl

Analytical Chemistry Group

Center for Analytical Sciences Amsterdam

Universiteit van Amsterdam

Science Park 904 |1098 XH Amsterdam

TABLE OF CONTENTS

**S1.0**	Chemicals and solutions	**S-3**
**Figure S1**	Schematic representation of the hyphenated platform based on AF4 coupled online to UV, MALS, dRI, and nMS	**S-4**
**Figure S2**	AF4-UV-MALS-dRI fractogram of ASNase	**S-4**
**Figure S3**	Fractograms of unstressed and stressed ASNase (A.) after agitation for 30 min and (B.) at 53 °C for 2 up to 24 h	**S-5**
**Figure S4**	(A.) Comparison of the elution profiles obtained for ASNase exposed to 10 mM NaOH at different the cross-flow rates. (B.) Comparison of the AF4 elution profile varying the injected amount	**S-5**
**Figure S5**	Fractogram of ASNase exposed to 10 mM NaOH for 8 h.	**S-6**
**Figure S6**	AF4-UV-dRI-MALS of ASNase stressed for 8 h with NaOH	**S-6**
**Figure S7**	Fractograms of ASNase using phosphate buffer and ammonium acetate	**S-7**
**Figure S8**	(A.) Sequence of L-Asparaginase from Escherichia coli. (B.) Simulated mass spectrum assuming one disulfide bridge between Cys77 and Cys105 is presented.	**S-7**
**Figure S9**	AF4-nMS of unstressed ASNase at varying in-source CID	**S-9**
**Figure S10**	AF4-UV-MS of ASNase exposed for 8 h to 10 mM NaOH	**S-10**
**Figure S11**	Fractograms of unstressed and stressed ASNase with 10 mg/mL ammonium bicarbonate	**S-11**
**Table S1**	m/z values used to construct the extracted ion chromatograms of the various ASNase oligomers	**S-8**

S1.0 Chemicals and solutions

The salts used for the preparation of the carrier liquid were purchased from Merck (Darmstadt, Germany). The phosphate-based carrier liquid (PB) consisted of 50 mM sodium chloride (NaCl), 25 mM sodium phosphate monobasic monohydrate (≥98%), 25 mM sodium phosphate dibasic dihydrate (≥99%), and 0.05% w/v sodium azide (≥99.5%). The carrier liquid used during AF4-nMS was 10 mM ammonium acetate (≥98%). Sodium hydroxide (≥98%, pellets, anhydrous) and ammonium bicarbonate (≥99%) were purchased from Sigma-Aldrich (Steinheim, Germany). All solutions were prepared using ultrapure water (resistivity 18.2 MΩ; Sartorius Arium 611UV; Sartorius, Göttingen, Germany). L-asparaginase (ASNase, Paronal, 10.000 I.U. equivalent to 40 mg powder per vial) produced in *E.coli* was obtained from Ghent University Hospital (Ghent Belgium). L-asparaginase was dissolved in the actual AF4 carrier liquid to a final concentration of 3 mg/mL, unless stated otherwise. The stressed samples were prepared by dissolving L-asparaginase with sodium hydroxide (10 mM) and ammonium bicarbonate (10 mg/mL). Bovine serum albumin (BSA; ≥96%) was purchased from Sigma-Aldrich.

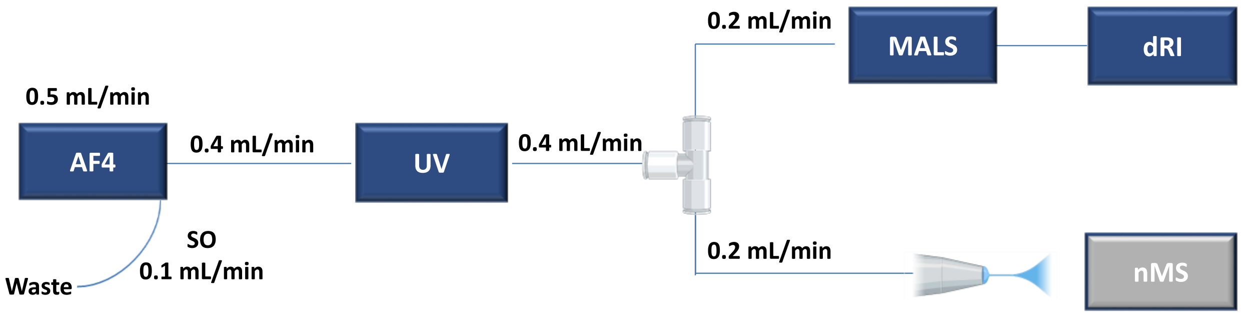

Figure S1. Schematic representation of the hyphenated platform based on AF4 coupled online to non-destructive non-destructive liquid-phase detectors (UV, MALS, dRI), and gas-phase nMS. The “slot-outlet” (SO) technique was used to reduce sample dilution at the channel outlet. A t-piece split with tubings of equal length (I.D. 0.25 mm) was connected to MALS-dRI and nMS.

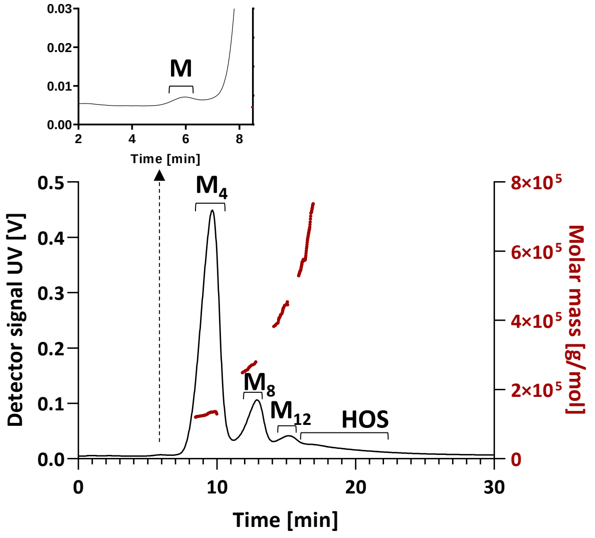

Figure S2. AF4-UV-MALS-dRI fractogram of ASNase. Carrier liquid:50 mM phosphate buffer, 50 mM sodium chloride (pH 6.8). F_c, 3.0 mL/min; F_{out}, 0.5 mL/min; injected amount, 30 µg. Peak annotation: M, monomer; M_4, tetramer; M_8, octamer; M_{12}, dodecamer; HOS, higher-order structures.

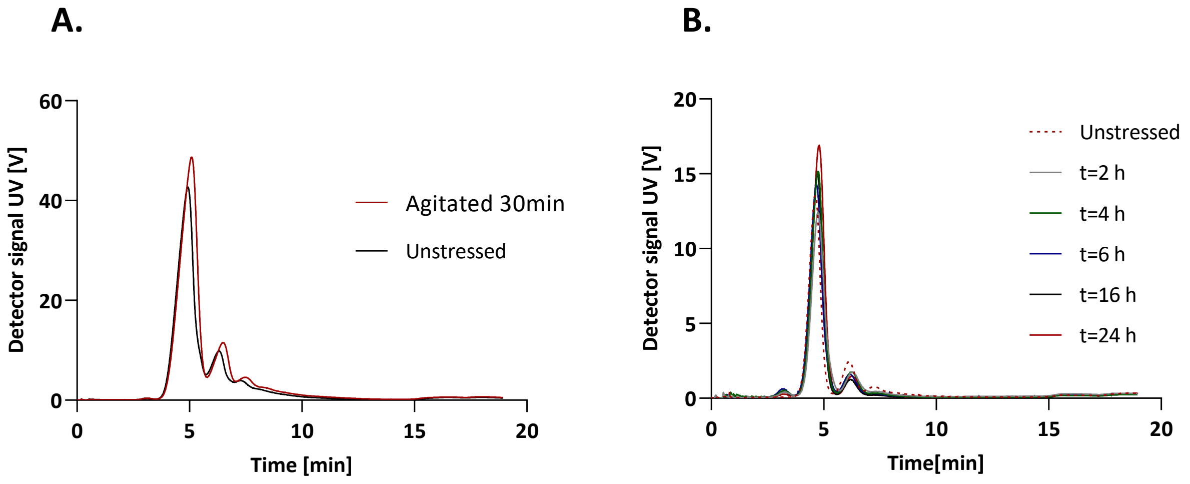

Figure S3 (A.) Comparison of the AF4 elution profile of unstressed ASNase (black trace) and after agitation for 30 min (red trace). (B.) Fractograms of unstressed and stressed ASNase at 53 °C for 2 up to 24 h. Injected amount was: 30 µg; carrier liquid: 50 mM phosphate buffer with 50 mM sodium chloride at pH 6.8. Constant F_c of 3.0 mL/min and F_{out} of 0.5 mL/min were used.

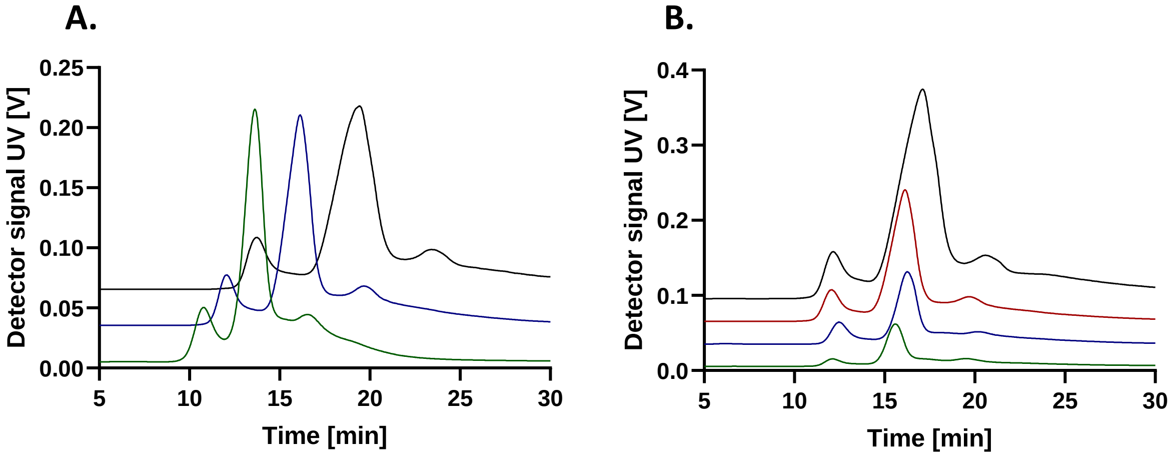

Figure S4. (A.) Comparison of the elution profiles obtained for ASNase exposed to 10 mM NaOH at different the cross-flow rates: 3.0 mL/min (green trace), 4.5 mL/min; blue, 5.5 mL/min (black trace), F_{out} was 0.5 mL/min. (B.) Comparison of the AF4 elution profile varying the injected amount 15 µg (green trace), 30 µg (blue trace), 60 µg (red trace), and 120 µg (black trace). Carrier liquid: 50 mM phosphate buffer with 50 mM sodium chloride at pH 6.8.

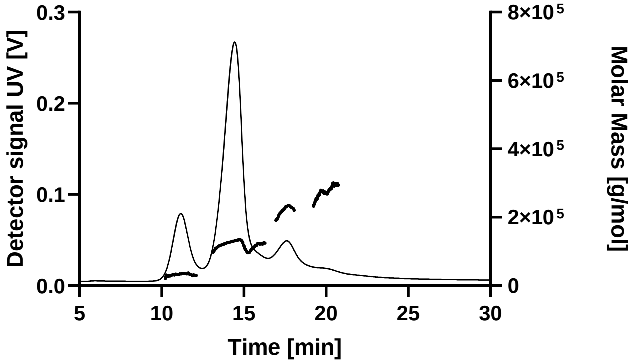

Figure S5. AF4 fractogram of ASNase exposed to 10 mM NaOH for 8 h. On the right-hand axis are the estimated molar masses at specific time points. Injected amount was 60 µg. Carrier liquid: 50 mM phosphate buffer with 50 mM sodium chloride at pH 6.8. Constant F_c of 3.0 mL/min and F_{out} of 0.5 mL/min were used.

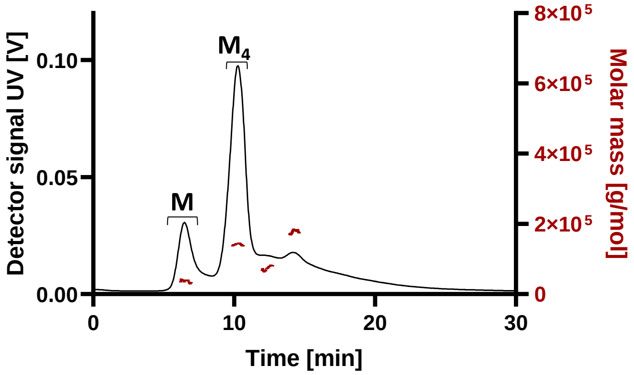

Figure S6. Fractogram of ASNase stressed for 8 h with NaOH. Injected amount: 30 µg; carrier liquid: 50 mM phosphate buffer with 50 mM sodium chloride at pH 6.8. Constant F_c of 4.5 mL/min and F_{out} = 0.5 mL/min were used.

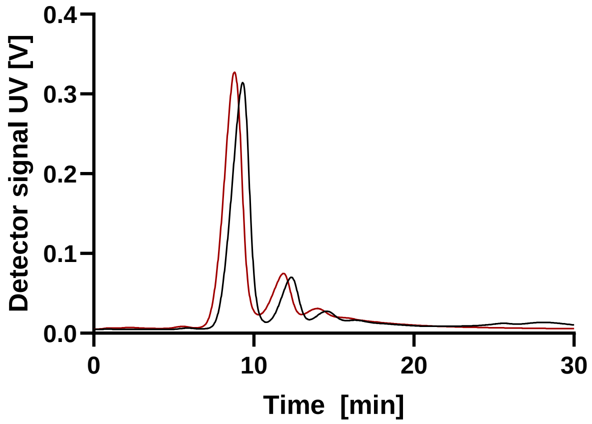

Figure S7. Comparison of the AF4 elution profiles of ASNase using 50 mM phosphate buffer with 50 mM sodium chloride (black trace) and 10 mM ammonium acetate (red trace) as carrier solvents. Constant F_c of 3.0 mL/min and F_{out} of 0.5 mL/min were used.

A. >sp|P00805|ASPG2_ECOLI L-asparaginase 2 OS=Escherichia coli (strain K12) OX=83333 GN=ansB PE=1 SV=2

MEFFKKTALAALVMGFSGAALALPNITILATGGTIAGGGDSATKSNYTVGKVGVENLVNA
VPQLKDIANVKGEQVVNIGSQDMNDNVWLTLAKKINTDCDKTDGFVITHGTDTMEETAYF
LDLTVKCDKPVVMVGAMRPSTSMSADGPFNLYNAVVTAADKASANRGVLVVMNDTVLDGR
DVTKTNTTDVATFKSVNYGPLGYIHNGKIDYQRTPARKHTSDTPFDVSKLNELPKVGIVY
NYANASDLPAKALVDAGYDGIVSAGVGNGNLYKSVFDTLATAAKTGTAVVRSSRVPTGAT
TQDAEVDDAKYGFVASGTLNPQKARVLLQLALTQTKDPQQIQQIFNQY

B.

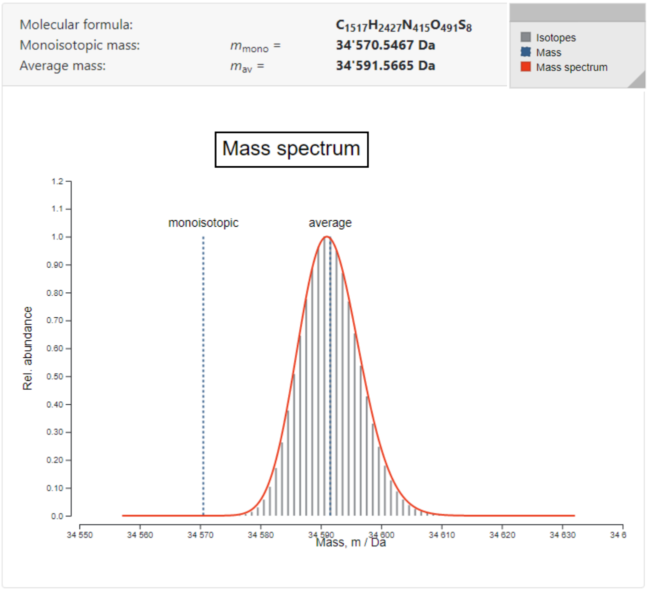

Figure S8. A) Amino acid sequence of L-Asparaginase from Escherichia coli. B) Simulated mass spectrum of ASNase monomer assuming one disulfide bridge between Cys77 and Cys105.

Table S1. The unique m/z values used to construct the extracted ion chromatograms of the various ASNase oligomers are highlighted in green. The overlapping m/z values are shown in red. The remaining m/z values were not present in the MS spectra. The relationship between the predicted maximum charge state and the theoretical mass of the oligomer (M) is calculated based on $z = 0.078 \cdot M^{1/2}$. The predicted m/z values are calculated based on the $(m/z)_z = \frac{M+Z}{Z}$ equation.

charge	monomer	dimer	trimer	tetramer	pentamer	hexamer	heptamer	octamer	nonamer	decamer
**6**	5766.17									
**7**	4942.57									
**8**	4324.88									
9	3844.44	7687.89								
10	3460.10	6919.20								
11	3145.64	6290.27								
12	2883.58	5766.17								
13	2661.85	5322.69	7983.54							
14	2471.79	4942.57	7413.36							
15		4613.13	6919.20							
16		4324.88	6486.81							
17		4070.53	6105.29							
18		3844.44	5766.17	7687.89						
19		3642.16	5462.74	7283.32						
20		3460.10	5189.65	6919.20						
21			4942.57	6589.76	8236.95					
22			4717.95	6290.27	7862.59					
23			4512.87	6016.83	7520.78					
24			4324.88	5766.17	7207.46					
25			4151.92	5535.56	6919.20					
26				5322.69	6653.12					
27				5125.59	6406.74	7687.89				
28					6177.96	7413.36				
29					5964.97	7157.76				
30					5766.17	6919.20				
31					5580.19	6696.03	7811.87			
32					5405.84	6486.81	7567.78			
33						6290.27	7338.48			
34						6105.29	7122.68			
35						5930.89	6919.20			
36							6727.03	7687.89		
37							6545.24	7480.14	8415.03	
38							6373.03	7283.32	8193.61	
39								7096.59	7983.54	
40								6919.20	7783.98	
41								6750.46	7594.15	**Too high for MS**
42									7413.36	8236.95
43									7240.98	8045.42
44										7862.59
45										7687.89

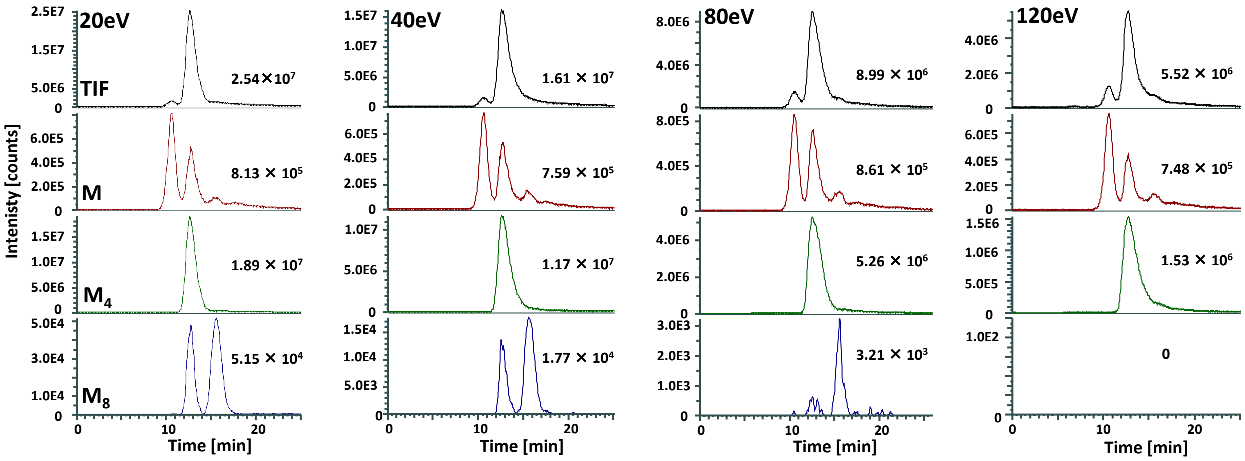

Figure S9. AF4-nMS of unstressed ASNase. Comparison of the total ion fractogram (TIF) and extracted ion chromatograms of the monomer (M), tetramer (M_4) and octamers (M_8) at varying in-source CID conditions (20, 40, 80, 120 eV). The intensity of the base peak is also reported.

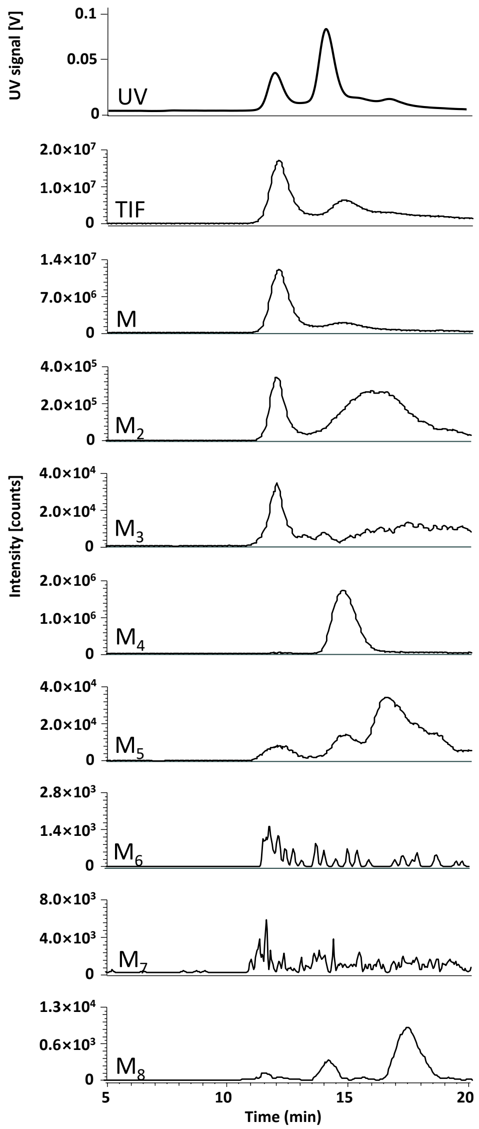

Figure S10. Analysis of ASNase exposed for 8 h to 10 mM NaOH by AF4-UV-MS. Top, UV fractogram; below, TIF and EIFs for various mono- and oligomeric species as generated using characteristic m/z values.

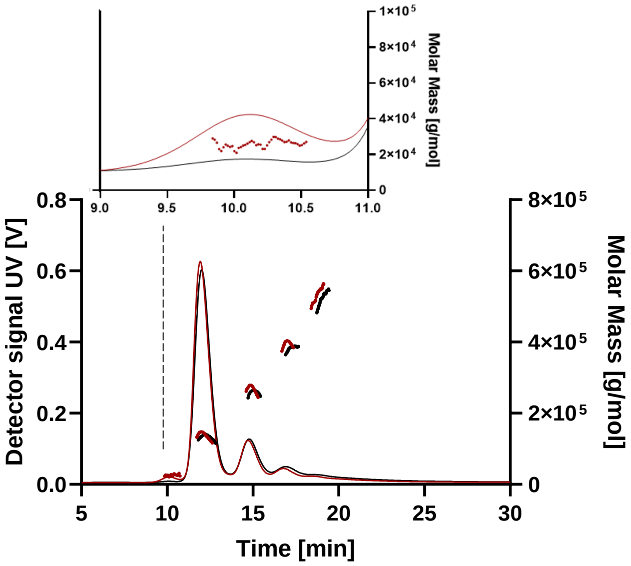

Figure S11. Overlaid AF4 fractograms of unstressed ASNase (black trace) and stressed with 10 mg/mL ammonium bicarbonate (red trace). Carrier liquid: 10 mM ammonium acetate (pH 6.9). A constant Fc of 3.0 mL/min was used and, Fout 0.4 mL/min and slot flow of 0.1 mL/min. Injected amount 60 µg. The flow was split (1:1) directing 0.2 mL/min to MALS-dRI and 0.2 mL/min to MS.
